# Interpreting the transmissibility of the avian influenza A(H7N9) infection from 2013 to 2015 in Zhejiang Province, China

**DOI:** 10.1017/S0950268815002812

**Published:** 2015-12-08

**Authors:** K. C. CHONG, X. WANG, S. LIU, J. CAI, X. SU, B. C. ZEE, G. TAM, M. H. WANG, E. CHEN

**Affiliations:** 1 JC School of Public Health and Primary Care, The Chinese University of Hong Kong, Hong Kong SAR, China; 2 Clinical Trials and Biostatistics Laboratory, Shenzhen Research Institute, The Chinese University of Hong Kong, Shenzhen, China; 3 Zhejiang Provincial Center for Disease Control and Prevention, Hangzhou, China

**Keywords:** Influenza A, mathematical modelling, Susceptible-Infected-Removed (SIR) model

## Abstract

Three epidemic waves of human influenza A(H7N9) were documented in several different provinces in China between 2013 and 2015. With limited understanding of the potential for human-to-human transmission, it was difficult to implement control measures efficiently or to inform the public adequately about the application of interventions. In this study, the human-to-human transmission rate for the epidemics that occurred between 2013 and 2015 in Zhejiang Province, China, was analysed. The reproduction number (*R*), a key indicator of transmission intensity, was estimated by fitting the number of infections from poultry to humans and from humans to humans into a mathematical model. The posterior mean *R* for human-to-human transmission was estimated to be 0·27, with a 95% credible interval of 0·14–0·44 for the first wave, whereas the posterior mean *R*s decreased to 0·15 in the second and third waves. Overall, these estimates indicate that a human H7N9 pandemic is unlikely to occur in Zhejiang. The reductions in the viral transmissibility and the number of poultry-transmitted infections after the first epidemic may be attributable to the various intervention measures taken, including changes in the extent of closures of live poultry markets.

## INTRODUCTION

Influenza A viruses are typically sustained in poultry and humans. Their transmission from avian to mammalian hosts is not common because their host ranges are restricted [1]. However, three large epidemic waves of human influenza A(H7N9) infection have been documented in China. The first human infections with H7N9 virus were reported in Shanghai, China, in February 2013. The number of confirmed cases increased exponentially in the eastern part of China, including Jiangsu and Zhejiang, and by the end of the first epidemic (27 July 2013), 135 confirmed cases of human infection with H7N9 virus (including 44 deaths) had been officially reported [2]. Of these 135 cases, 46 were residents of Zhejiang Province, representing the highest percentage of cases on mainland China, and 11 were fatal. Although there were no further cases in the following 2 months, human infections with avian influenza A(H7N9) virus re-emerged in Zhejiang Province on 14 October 2013 [3]. The laboratory-confirmed cases gradually increased to 93 human cases, including 39 deaths, with the last case reported in February, 2014 [4]. The third epidemic wave began shortly thereafter, in November 2014. Compared with the first epidemic wave, the later waves included larger numbers of human H7N9 infections and spread across wider geographical areas. Furthermore, most cases that arose in the later waves were identified in the southern provinces of China, such as Guangdong.

In an epidemic, the reproduction number (*R*) is typically used to measure the transmission potential of an infectious disease. *R*, defined as the average number of secondary infections produced by a typical infected individual, is a key parameter in quantifying the transmission intensity in a population. The quantity *R* can be used to determine the intensity of interventions that should be applied, and *R* must be maintained at <1 to prevent a pandemic. If *R* is large, more control measures and interventions should be introduced in the community.

According to a risk assessment conducted by the World Health Organization [5], the avian influenza A(H7N9) virus is not easily transmitted among humans. However, limited human-to-human transmission may occur when individuals are not protected from close contacts with symptomatic disease. Chowell *et al*. [6] and Nishiura *et al*. [7] have used mathematical modelling to estimate the values for *R* in order to investigate the transmission potential of the first outbreak of influenza A(H7N9), in 2013. Other successive modelling studies [8, 9] provided additional insight into the first wave of the epidemic. All their findings indicated that a pandemic of the novel influenza A(H7N9) was unlikely to occur because its human-to-human transmission potential was low (*R* <1). Because this was the first wave of the human epidemic, our limited understanding of the virus and its mode of transmission made it difficult to implement effective control measures, and the public was confused about the use of interventions such as vaccination. The calculation of *R* in successive waves allowed the effects of control measures on the transmission intensity of the virus to be assessed.

In this study, we investigated the human-to-human transmission potential of the epidemic of a novel influenza A(H7N9) in Zhejiang Province, China between 2013 and 2015, based on laboratory-confirmed data collected from the Zhejiang Provincial Center for Disease Control and Prevention (CDC). We particularly examined the differences in human-to-human transmissibility in the three epidemic waves.

## METHODS

### Data

**Fig. 1.**
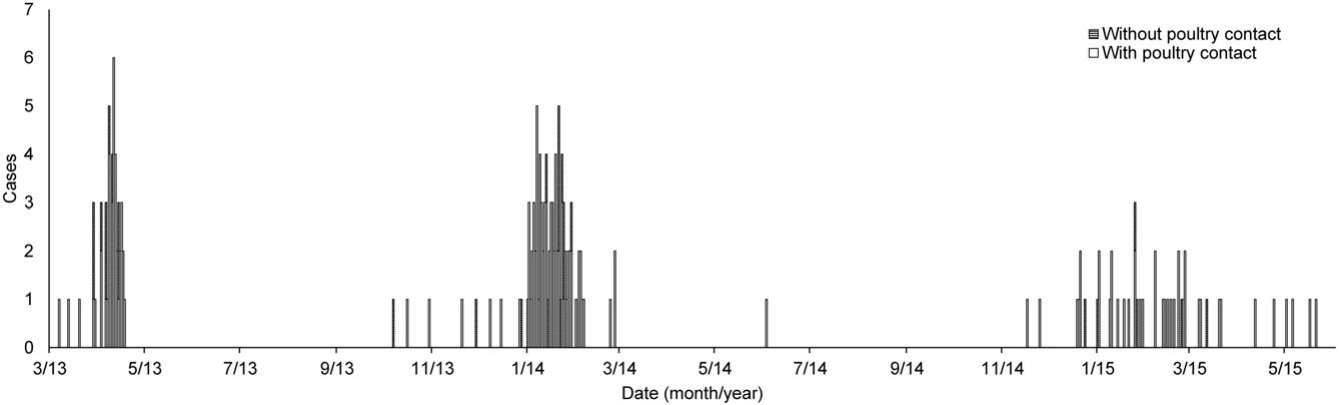
Temporal distribution of avian influenza A(H7N9) cases in the Zhejiang Province from 2013 to 2015.

Three seasons of an avian influenza A(H7N9) epidemic (March 2013–April 2013, October 2013–February 2014, November 2014–May 2015) in Zhejiang Province were analysed in this study. Laboratory-confirmed patients who were infected with H7N9 virus were identified according to the guidelines issued by the National Health and Family Planning Commission of the People's Republic of China [10, 11]. The details of the laboratory tests have been described previously [12]. The exposure histories of the patients, such as visits to live poultry markets and direct contact with poultry, were collected with in-depth interviews. A plot of the daily incidence of infection is given in Figure 1. The first cases of illness became apparent on 7 March 2013, 7 October 2013, and 17 November 2014 in the three epidemic waves, with 46, 93, and 46 total cases, respectively. Overall, around 85% of cases had a history of exposure to poultry.

### Transmission model

The development of the likelihood function was based on based on the number of cases determined with the Kermack & McKendrick model [13]. The model classifies a whole population of size *N* into one of three compartments at each time point *t* (*t* = 0, 1, 2, 3, …): susceptible [*S*(*t*)], infectious [*I*(*t*)], or recovered. In the model, a susceptible individual can be infected by either poultry or a human. Let *λ*_*A*_(*t*) be the number of cases infected by exposure to poultry and *βS*(*t*)*I*(*t*)*/N* be the number of cases caused by human-to-human transmission, given the transmission rate *β.* The Kermack & McKendrick model can be described with the following differential equations, which define the rates of subject movement at each time step:

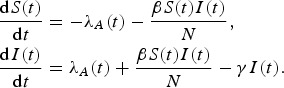

**Fig. 2.**
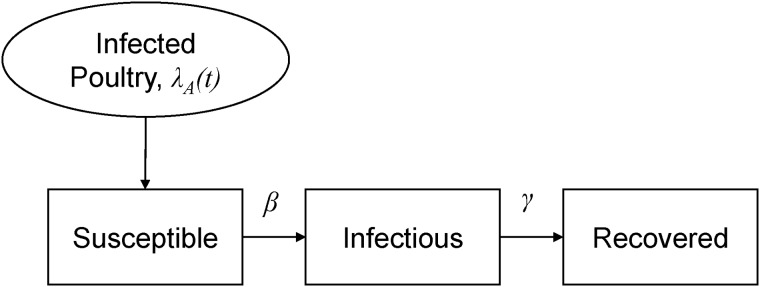
Schematic flow of the transmission model for influenza A(H7N9).

The schematic flow of the transmission model is shown in Figure 2. During the initial phase of a new epidemic, the number of susceptibles is similar to the total population *N, S*(*t*) *≈* N, assuming there is no prior immunity among the individuals. Given an exponential assumption, the length of the infectious period is equal to 1/*γ*, and the reproduction number *R* is always equal to *β/γ* [14]. Therefore, the expected number of cases caused by human-to-human transmission would be:

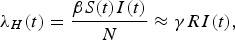

Because the number of cases was small, we assumed a constant parameter for the generation function for cases infected by exposure to poultry, *λ*_*A*_(*t*) = *λ*_*A*_. The parameters *R* and *λ*_*A*_ were estimated using the Markov Chain Monte Carlo (MCMC) method in a Bayesian inferential framework.

### Statistical inference

We estimated the model parameters based on the time-series data for the symptom onset dates for the confirmed cases infected by poultry-to-human transmission or by human-to-human transmission. To this end, we developed a likelihood function from the transmission model by assuming Poisson-distributed outcomes:

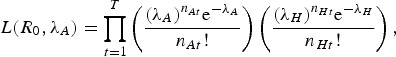


where *T* is the number of days for which onset data were available. *λ*_*A*_ and *n*_*At*_ are the expected and observed numbers of cases infected by exposure to poultry on day *t*, respectively. *λ*_*H*_ and *n*_*Ht*_ are the expected and observed numbers of cases infected by human-to-human transmission on day *t*, respectively.

### Estimation

An MCMC approach was used to estimate the model parameters. We assumed flat prior distributions for *R* and *λ*_*A*_, with ranges of 0·001–2 and 0·01–20, respectively. Based on the estimate from the Zhejiang CDC data, the mean infectious period (1/*γ*) was fixed at 10 days. The mean infectious period was comparatively longer than in other studies, because the clinical status of the reported cases was more severe. The random walk Metropolis algorithm was used to obtain the posterior distributions of the parameters. The step sizes were selected to obtain acceptance proportions between 20% and 40%. A total of 200 000 MCMC iterations were used as the burn-in period, and the subsequent 800 000 iterations were used to obtain the estimates. The convergence of the Markov chain mixing in the MCMC process was diagnosed with the autocorrelation function and a time-series trace plot. Stationary chains represent a good mixing pattern in MCMC. The means, standard deviations (s.d.), and 95% credible intervals (CrIs) of the posterior distributions were calculated to summarize the estimates.

### Sensitivity analysis

To account for the overdispersed incidence, we also considered the negative binomial (NB) distributions in the construction of the likelihood function. The variance of the distribution was set to double the mean. We also assumed a shorter and a longer infectious period of 5 days and 15 days, respectively. Moderate and severe situations of underreporting were assumed by fixing the reporting rate (*r*) to 10% and 50%, respectively. Sensitivity was assessed by replacing the number of observed cases with their values divided by the reporting rate in the estimation, *n*_*At*_/*r* and *n*_*Ht*_/*r*. The days with zero cases were interpolated from the number of cases in the preceding and subsequent days.

## RESULTS

**Fig. 3.**
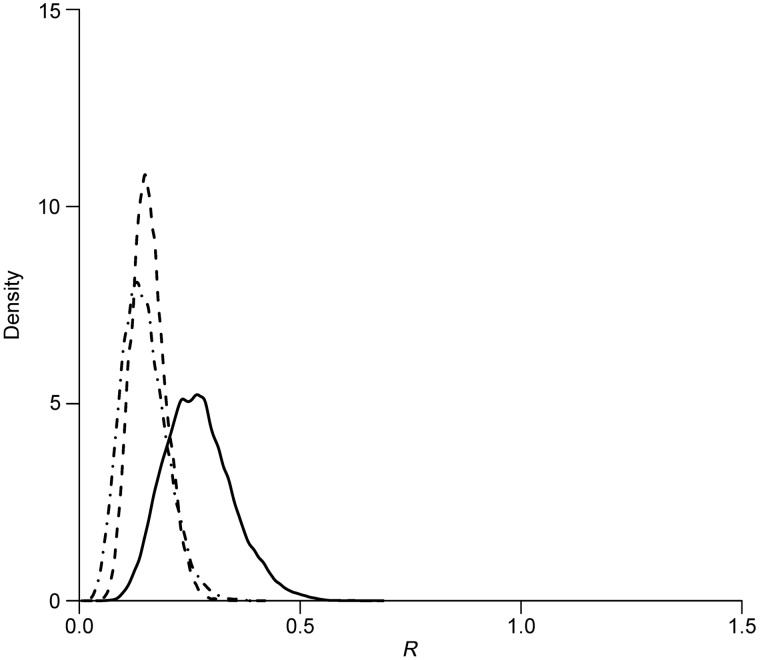
Density plot of the posterior distributions of reproduction numbers for the first (solid line), second (dashed line), and third (dot-dashed line) waves of influenza A(H7N9) epidemics.

The convergences of the MCMC iterations were obtained to ensure the existence of a stationary distribution of the estimates. Table 1 summarizes the estimates. For the first wave of the epidemic, the posterior estimate of mean *R* for human-to-human transmission was 0·27 (95% CrI 0·14–0·44). The posterior distribution of the estimate is shown in Figure 3. The posterior mean number of cases caused by poultry-to-human transmission (*λ*_*A*_) was estimated to be 0·87 (95% CrI 0·62–1·17). For the second and third waves, the posterior mean *R*s were estimated to be around 0·15, with 95% CrIs of 0·09–0·24 and 0·06–0·26, respectively. Both estimates were lower than that of the first wave (Fig. 3). The posterior means of *λ*_*A*_ were estimated to be 0·56 (95% CrI 0·44–0·68) and 0·22 (95% CrI 0·15–0·29) in the second and third waves, respectively. The upper bound of the 95% CrI of *R* for all epidemic waves was <1.

**Table 1.** Summary statistics of posterior distributions generated by the Markov Chain Monte Carlo method for human-to-human transmission during three waves of influenza A(H7N9) epidemics in Zhejiang Province, China

	First wave		Second wave		Third wave	
Parameter	Mean (s.d.)	95% CrI	Mean (s.d.)	95% CrI	Mean (s.d.)	95% CrI
*R*	0·27 (0·08)	0·14–0·44	0·15 (0·04)	0·09–0·24	0·15 (0·05)	0·06–0·26
*λ*_*A*_	0·87 (0·14)	0·62–1·17	0·56 (0·06)	0·44–0·68	0·22 (0·03)	0·15–0·29

CrI, Credible interval; *R*, reproduction number; *λ*_*A*_, mean number of cases generated by poultry-to-human transmission.

**Fig. 4.**
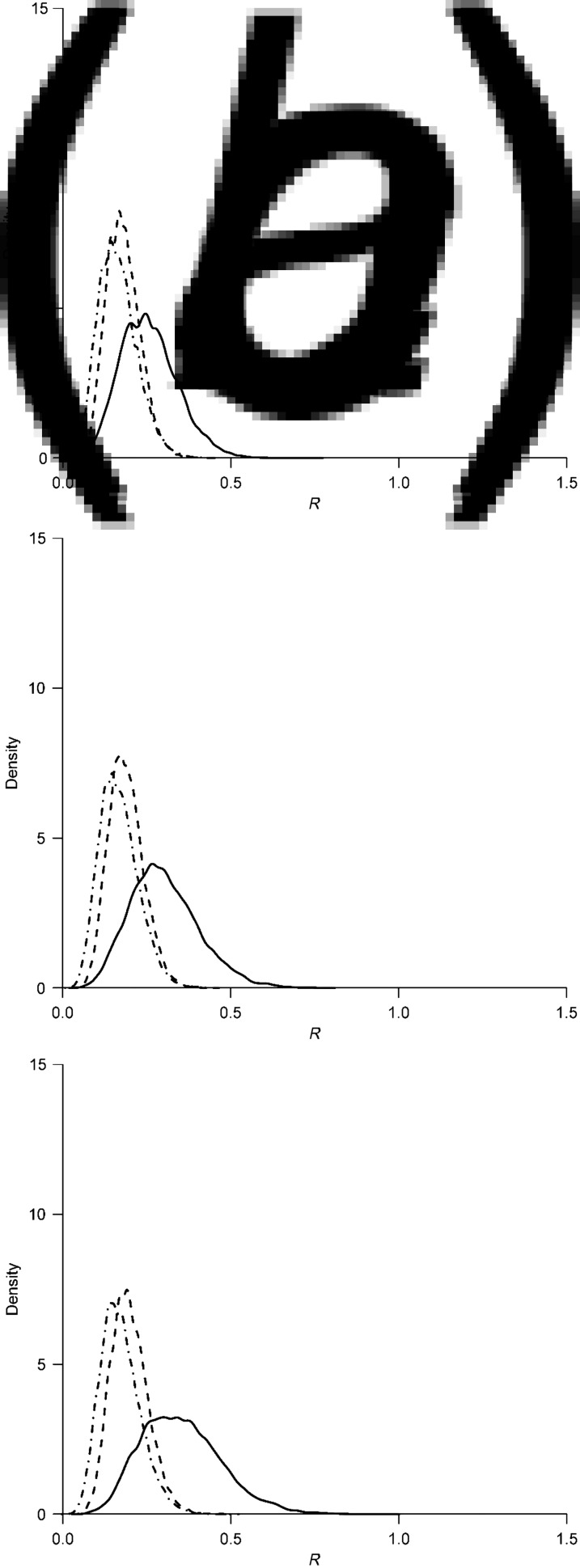
Density plot of the posterior distributions of reproduction numbers when incidence followed negative binomial distribution with duration of infectiousness fixed as (*a*) 5 days, (*b*) 10 days, and (*c*) 15 days, respectively for the first (solid line), second (dashed line), and third (dot-dashed line) waves of influenza A(H7N9) epidemics.

A sensitivity analysis was conducted to assess the robustness of the parameter estimates. Tables 2 and 3 summarize the results of the sensitivity analysis. Our estimates were robust to the NB distributional assumption that was used to explain the dispersed data. With a fixed infectious duration of 10 days, the posterior mean *R* increased slightly to 0·30 (95% CrI 0·13–0·53), 0·19 (95% CrI 0·10–0·30), and 0·17 (95% CrI 0·07–0·30) for the three epidemics, respectively, compared to the estimates based on the Poisson assumption (Fig. 4). In addition to the NB distribution assumption, we also tested the effects of a shorter infectious period (5 days) and a longer infectious period (15 days) on our results. Variations in the infectious period did not change the estimates of *λ*_*A*_. The *R* estimate was also robust to the variations in the infectious period. The results were similar when the length of the infectious period was varied, i.e. the ranges of the mean estimates were 0·26–0·35, 0·18–0·20, and 0·16–0·18 for the three epidemics, respectively, when the infectious period was equal to 5 days or 15 days (Fig. 4).

**Table 2.** Summary statistics for estimates with different lengths of infectious duration

		First wave		Second wave		Third wave	
Settings*	Parameter	Mean (s.d.)	95% CrI	Mean (s.d.)	95% CrI	Mean (s.d.)	95% CrI
Infectious duration (5 days)	*R*	0·26 (0·08)	0·11–0·44	0·18 (0·05)	0·10–0·29	0·16 (0·06)	0·07–0·29
	*λ*_*A*_	0·83 (0·18)	0·52–1·23	0·51 (0·08)	0·37–0·67	0·27 (0·05)	0·19–0·37
Infectious duration (10 days)	*R*	0·30 (0·10)	0·13–0·53	0·19 (0·05)	0·10–0·30	0·17 (0·06)	0·07–0·30
	*λ*_*A*_	0·83 (0·18)	0·52–1·22	0·51 (0·08)	0·37–0·68	0·27 (0·05)	0·19–0·37
Infectious duration (15 days)	*R*	0·35 (0·12)	0·15–0·62	0·20 (0·05)	0·10–0·31	0·17 (0·06)	0·07–0·30
	*λ*_*A*_	0·83 (0·18)	0·51–1·22	0·51 (0·08)	0·37–0·68	0·27 (0·05)	0·19–0·37

CrI, Credible interval; *R*, reproduction number; *λ*_*A*_, mean number of cases generated by poultry-to-human transmission.

* Negative binomial distribution was assumed for the generation of the incidence.

**Table 3.** Summary statistics for estimates with different reporting rates

		First wave		Second wave		Third wave	
Settings	Parameter	Mean (s.d.)	95% CrI	Mean (s.d.)	95% CrI	Mean (s.d.)	95% CrI
*r* = 50%	*R*	0·37 (0·03)	0·32–0·43	0·30 (0·02)	0·28–0·33	0·28 (0·02)	0·26–0·31
	*λ*_*A*_	7·63 (0·42)	6·81–8·51	6·18 (0·21)	5·78–6·61	5·89 (0·18)	5·57–6·27
*r* = 10%	*R*	0·51 (0·04)	0·47–0·55	0·37 (0·02)	0·34–0·39	0·32 (0·02)	0·30–0·34
	*λ*_*A*_	9·96 (0·05)	9·84–10·0	9·97 (0·03)	9·88–10·0	9·97 (0·03)	9·90–10·0

CrI, Credible interval; *r*, reporting rate; *R*, reproduction number; *λ*_*A*_, mean number of cases generated by poultry-to-human transmission.

**Fig. 5.**
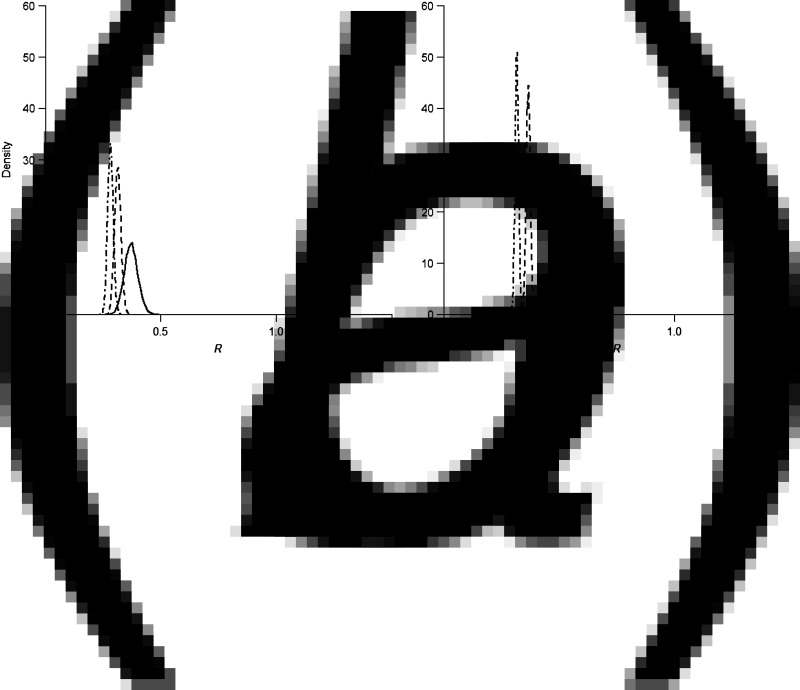
Density plot of the posterior distributions of reproduction numbers when reporting rate fixed as (*a*) 50% and (*b*) 10%, respectively, for the first (solid line), second (dashed line), and third (dot-dashed line) waves of influenza A(H7N9) epidemics.

When the reporting rate dropped to 50%, *R* clearly increased to 0·37 (95% CrI 0·32–0·43), 0·30 (95% CrI 0·28–0·33), and 0·28 (95% CrI 0·26–0·31) for the three epidemics, respectively. The estimates increased twofold when the reporting rate was only 10% (Fig. 5). However, adjustment of the reporting rate did not cause other conclusions to be drawn about the pandemic potential of the virus or the pattern of human-to-human transmissibility over the years. In brief, the estimates of the parameters were not sensitive to either the NB distribution, the length of infectious period, or the reporting rate.

## DISCUSSION

We used a simple Kermack & McKendrick SIR model to estimate the reproduction number *R* for the epidemic of the novel influenza A(H7N9) in Zhejiang Province, China, from 2013 to 2015, based on laboratory-confirmed data from the Zhejiang Provincial CDC. From our results, the estimates of *R* for human-to-human transmission were <1 for all three epidemic waves, indicating that an avian influenza A(H7N9) pandemic was unlikely to occur in Zhejiang. These estimates were consistent with those of other similar studies that investigated the transmissibility in the first waves of this and other epidemics [6–9] (Table 4). In two of these studies, Chowell *et al.* [6] estimated the *R* of human-to-human transmission of the virus in Zhejiang to be 0·13 and Kucharski *et al.* [8] estimated it to be 0·03. Our estimate was slightly greater than theirs, which may be because different infectious periods and serial intervals were used. The *R* estimates of Nishiura *et al*. [7] and Xiao *et al*. [9] were greater than those cited above, and Xiao *et al.* [9] even estimated the value to be 0·47 (95% CrI 0·39–0·65). However, all the estimates were <1, indicating that a human-to-human pandemic was unlikely to occur.

**Table 4.** Brief review of reproduction numbers for the first wave of influenza A(H7N9) epidemics

*R* estimate	Reference
Shanghai: 0·15 (95% CrI 0·01–0·47) Zhejiang: 0·13 (95% CrI 0·01–0·46)	[6]
0·28 (95% CI 0·11–0·45)	[7]
Shanghai: 0·19 (95% CrI 0·01–0·49) Jiangsu: 0·29 (95% CrI 0·03–0·73) Zhejiang: 0·03 (95% CrI 0·00–0·22)	[8]
0·47 (95% CI 0·39–0·65)	[9]

CrI, Credible interval; CI, confidence interval.

In this study, the estimated human-to-human transmissibility and the mean number of cases caused by poultry-to-human transmission decreased over time. Several factors may have contributed to these reductions. First, the implementation of mitigation measures improved after the first wave of the H7N9 epidemic, including the increased extent and duration of live poultry market closures, because our understanding of the severity and transmissibility of the virus had increased. During the first epidemic wave, around 29·5% of towns in Zhejiang (including all the towns with reported cases of infection) closed their live poultry markets. When the second epidemic occurred, this proportion increased to >70%. Based on these experiences, officials closed all the live poultry markets of the central towns in July 2014 to prevent a potential outbreak of the third H7N9 epidemic. The epidemics were thus alleviated, although some individuals infected from rural markets or illegally trading markets were still reported [15]. Second, most cases in the first H7N9 epidemic occurred in spring, whereas the later epidemics occurred in late winter in Zhejiang. Moreover, more cases were identified in the southern provinces of China, such as Guangdong, in the later epidemics. These trends may have been associated with the migration patterns of birds, which are believed to be the ultimate source of the virus [16]. Several other factors, including human mobility [17] and meteorological parameters [18], may also have been associated with the reduction in *R*, although the difference in temperature between late-winter and spring in Zhejiang Province is only around 5–8 °C, and there is almost no difference in average humidity. These effects on the H7N9 transmission potential warrant further investigation with better planned data collection.

To date, many studies have shown that most H7N9 cases were poultry-to-human transmission, with very limited instances of human-to-human transmission [6–9, 19]. In our study, we did not estimate the transmissibility from poultry to humans [16, 20, 21] because collecting information on poultry populations, such as population size, density, and number of poultry infections, is difficult [22]. Although the poultry-to-human infection rate could be an important indicator of the intensity of poultry market disinfection, the variety of bird types involved, including migrant birds and resident birds, and the large-scale migration of migrant birds will make the disease harder to control. Zhang *et al.* even suggested that the original infection source of H7N9 was migrant birds or resident birds [16]. In China, most domestic poultry are usually kept in outdoor environments and poultry farms are very exposed to other wild birds. Therefore, infected migrant birds could be the source of infection, contaminating poultry farms. According to Zhang *et al.* [16], domestic poultry alone are unlikely to be the original infection source. Indeed, we found that some environmental samples were positive for H7N9 when tested by the CDC laboratory (results of another study). Although there is no solid evidence, this is a likely cause of the reappearance of the disease, even though almost all live poultry trading markets had been closed.

One of the limitations of our study is that we did not parameterize the control measures, such as the closure of the poultry markets, in the mathematical model [9, 23] because the sampling data for poultry infections were limited. This made it difficult to identify the factors associated with the changes in *R*. A further investigation is required to evaluate the impact of control measures on the human-to-human transmissibility of the virus. Another limitation was that patients with a known history of contact with poultry were all assumed to have been infected by poultry-to-human transmission in our estimation method. This assumption would undoubtedly have been violated because they may also have been exposed to infected humans, such as family members. However, tracing routine contacts maximized the detection of instances of human-to-human transmission. Another major limitation of our study was the assumption of homogeneous mixing, so that population characteristics, such as age and sex, were not incorporated into our model. The limited numbers of confirmed cases made the assumption of heterogeneity difficult to adopt.

In conclusion, we used the onset data for a novel influenza A(H7N9) illness to estimate the human-to-human transmissibility of the virus during epidemics in Zhejiang Province, China. Based on our estimates, we found that the transmissibility and poultry-to-human transmission decreased after the first epidemic outbreak. These reductions may be attributable to several factors, including changes in the closure of live poultry markets based on the experience of the initial epidemic.
